# Type I interferon-dependent IFIT3 signaling is critical for viral clearance in airway neutrophils

**DOI:** 10.21203/rs.3.rs-1812836/v1

**Published:** 2023-03-21

**Authors:** Camilla Margaroli, Timothy Fram, Nirmal S. Sharma, Siddharth B. Patel, Jennifer Tipper, Sarah W. Robison, Derek W. Russell, Seth D. Fortmann, Mudassir M. Banday, Tarek Abdalla, Sawanan Saitornuang, Matthew C. Madison, Sixto M. Leal, Kevin S. Harrod, Nathaniel B. Erdmann, Amit Gaggar

**Affiliations:** 1 Department of Medicine, Division of Pulmonary, Allergy & Critical Care Medicine, University of Alabama at Birmingham, Birmingham, Alabama, USA.; 2 Program in Protease and Matrix Biology, University of Alabama at Birmingham, Birmingham, Alabama, USA.; 3 Department of Medicine, Division of Infectious Diseases, University of Alabama at Birmingham, Birmingham, Alabama, USA.; 4 Department of Medicine, Division of Pulmonary, Allergy, and Critical Care Medicine, Brigham and Women’s Hospital, Boston, Massachusetts, USA; 5 Department of Anesthesiology and Perioperative Medicine, University of Alabama at Birmingham, Birmingham, Alabama, USA.; 6 Department of Ophthamology, University of Alabama at Birmingham, Birmingham, Alabama, USA.; 7 Department of Pathology, Division of Laboratory Medicine, University of Alabama at Birmingham, Birmingham, Alabama, USA.; 8 Lung Health Center and Gregory Fleming James CF Center, University of Alabama at Birmingham, Birmingham, Alabama, USA.; 9 Birmingham VA Medical Center, Birmingham, Alabama, USA.

## Abstract

Neutrophilic inflammation characterizes several respiratory viral infections including COVID-19-related ARDS, although its contribution to disease pathogenesis remains poorly understood. Here, we identified two neutrophil subpopulations (A1 and A2) in the airway compartment of 52 severe COVID-19 subjects, where loss of the A2 subset correlated with increased viral burden and reduced 30-days survival. A2 neutrophils showcased a discrete antiviral response with an increased interferon signature. Blockade of type I interferon attenuated viral clearance in A2 neutrophils and downregulated IFIT3 and key catabolic genes, demonstrating direct antiviral neutrophil function. Knockdown of IFIT3 in A2 neutrophils led to loss of IRF3 phosphorylation with consequent reduced viral catabolism, providing the first discrete mechanism of type I interferon signaling in neutrophils. The identification of this novel neutrophil phenotype and its association with severe COVID-19 outcomes emphasizes its likely importance in other respiratory viral infections and potential for new therapeutic approaches in viral illness.

Viral respiratory infections are a major cause of worldwide morbidity and mortality ^1^, as recently evidenced by the COVID-19 pandemic. Severe COVID-19 presentations are characterized by respiratory failure and acute respiratory distress syndrome (ARDS)^2^. Robust neutrophilic inflammation characterizes several respiratory viral infections ^3^ including COVID-19-related ARDS ^4^. An increased number of circulating monocytes and neutrophils have been reported in SARS-CoV-2 infection ^5,6^ and studies in early-stage COVID-19 patients identified a high neutrophil-to-lymphocyte ratio to be a biomarker for disease progression ^4^. Differences in myeloid cell activation in blood at the time of hospitalization have been correlated with disease severity ^7,8^, with systemic neutrophil activation in blood at the time of hospitalization correlating with the development of the most severe COVID-19 cases ^8^.

While the adaptive immune response plays a key role in viral immunity, the role of neutrophils in modulating the inflammatory landscape in viral lung disease such as COVID-19 and their contributions to clinical disease heterogeneity remain poorly defined. Neutrophil heterogeneity is increasingly recognized as a critical regulator of inflammatory disorders ^9^ but remains poorly understood in viral pathogenesis. Neutrophil subpopulations expressing interferon stimulated genes (ISGs) have been previously reported in the peripheral blood and spleen at homeostasis, during bacterial infections ^10^ and in the tumor microenvironment ^11^. More recently, ISG-expressing neutrophils were found in the blood of severe COVID-19 patients ^12,13^ although functional implications of these gene signatures has not been shown. Here, we investigated presence of these neutrophil subpopulations in the airways of severe COVID-19 subjects and determined the functional impact of type I interferon signaling in airway neutrophils.

## Results:

### Airway neutrophil phenotype discriminates patient survival

**Figure 1.**
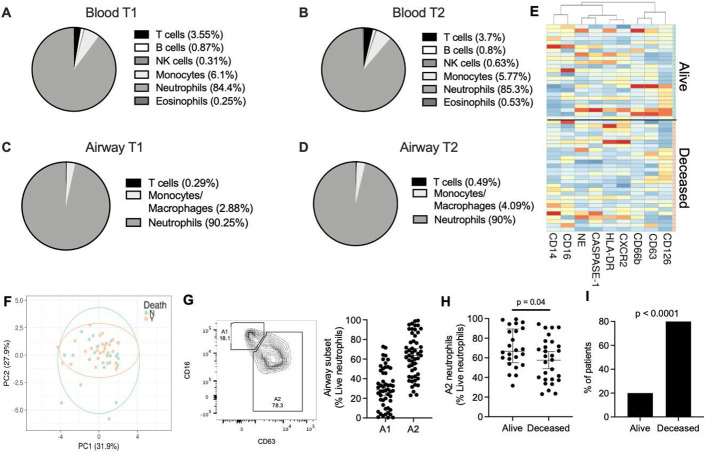
Airway neutrophil subsets associate with survival. Blood and airway immune cell frequencies (live and CD45+) and profiles were determined by flow cytometry. COVID-19 patients displayed blood neutrophilia **(A)** upon ICU admission (T1, n = 52) (Normal neutrophil frequencies: 40-65%). These profiles were maintained at time point 2 (T2, n = 28) **(B)**. Airway immune cell frequencies in mBAL displayed marked neutrophil infiltration, which was maintained through both time points **(C-D)**. **(E)** No significant difference was observed between surviving and deceased patient for individual surface markers, or as a combined profile by principal component analysis **(F)**. **(G)** Presence of specific neutrophil subsets, including airway neutrophils profiles matching the A1 and A2 populations. **(H)** A2 neutrophil frequency at time of admission discriminated 30-day mortality (Alive =28 , Deceased =24). **(I)** Low frequencies of the A2 population correlated with mortality (Alive =3 , Deceased =10). Results are shown as median and interquartile range in G and H. Statistical analysis was performed using the unpaired t-test upon normality testing and the Fisher’s exact test for unpaired analysis.

We initially investigated innate immune cell frequencies and phenotypes systemically and locally in the lung, and how these profiles changed over time. Blood and airway immune cells from 52 COVID-19 patients requiring intensive care and mechanical ventilation **(Table S1)** were collected within 3 days of intubation and patients were resampled again 7 days later. Analysis by flow cytometry **(Fig. S1)** showed that COVID-19 patients displayed marked blood neutrophilia upon ICU admission (compared to normal neutrophil frequencies: 45-65% of CD45+), and low T cell and monocyte frequencies (Fig. 1A). No significant differences were observed in blood neutrophils, monocytes, or T cell frequencies over the two measured time points (Fig. 1B).

Next, we investigated whether neutrophil frequencies in the peripheral circulation were mirrored in the lung. Similar to the systemic profiles, the airway immune cell landscape displayed a predominance of neutrophils which remained consistent over the two time points (Fig. 1C and 1D). Neutrophil frequencies in blood did not statistically correlate with airways at first time point **(Fig. S2A)**, but did at time point 2 **(Fig. S2B)**, and no correlation was found longitudinally between blood at time point 1 and airways at time point 2 **(Fig. S2C)**. Further, no difference was observed in neutrophil frequencies (systemic or lung) with 30-day survival (28 alive; 24 deceased) at neither time point, nor was there a difference present longitudinally within each group **(Fig. S2D)**. Neutrophil frequency in recovering patients did not correlate with disease severity as defined by length of ICU admission (Rho= −0.254), intubation time (Rho= −0.265), hospitalization time (Rho= −0.334), or APACHE II at time of admission (Rho = −0.455). Likewise, activation profiles of airway neutrophils did not show significant differences over time **(Fig. S2E-I)**, and it did not discriminate survival in our 52 patient cohort, either as individual markers (Fig. 1E) or in combination by principal component analysis (Fig. 1F).

Given the differential neutrophil activation observed in other forms of ARDS ^14^ we performed a clustering analysis (Uniform Manifold Approximation and Projection for Dimension Reduction – UMAP, as detailed in the methods section) of airway neutrophils in our cohort of COVID-19 patients. UMAP analysis revealed the presence of distinct airway neutrophil subsets (A1 and A2), defined by the loss of surface CD16 and release of primary granules as measured by surface CD63 **(Fig. S2J and 1G, left panel)**. These subsets have been previously described in cystic fibrosis airways, with the A2 population entailing phenotypic, metabolic, and transcriptional differences, although the biological role of these cells is unclear ^15-17^. While A1 and A2 frequencies did not differ over time **(Fig. S2K)**, and the delta between time point 2 and time point 1 did not discriminate any of the measured clinical parameters, several patients displayed a marked difference in the frequencies of A1 and A2 at time point 1 (Fig. 1G**, right panel**), prompting us to investigate whether their frequencies correlated with patient outcomes at 30 days post-admission to the ICU. Interestingly, ICU patients who survived to 30 days post-admission displayed a neutrophil activation profile skewed towards that of the A2 population (Fig. 1H). Indeed, lower frequencies of the A2 population correlated with increased mortality (Fig. 1I), suggesting that the A2 neutrophil phenotype may be related with different disease dynamics in COVID-19 patients.

### A2 neutrophils exhibit antiviral transcriptional signatures with increased type I interferon

**Figure 2.**
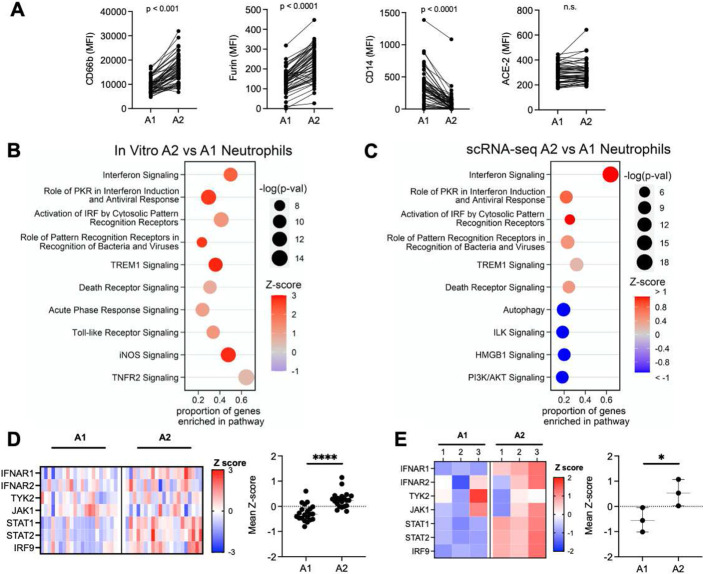
A2 neutrophils show antiviral gene signatures. **(A)** Airway neutrophils were profiled by flow cytometry at time point 1 (n = 52). A1 and A2 neutrophils expression of surface CD66b, CD14, furin and ACE-2 (MFI: median fluorescence intensity). **(B)** Pathway analysis for genes enriched in A2 neutrophils generated *in vitro* (n=3 donors). **(C)** Pathway analysis for genes enriched in A2 vs A1 BAL neutrophils from scRNA-seq (n=21 patients). **(D)** Type I interferon pathway gene expression for A2 vs A1 BAL neutrophils from scRNA-seq with mean z-score (n=21 patients). **(E)** Type I interferon pathway gene expression for A2 vs A1 neutrophils generated *ex vivo* mean z-score (n=3 per group). Data are shown as median and interquartile range. Statistical analysis was performed using Wilcoxon matched-pair signed rank’s test for paired analysis, Wilcoxon rank-sum test for unpaired analysis. * p<0.05; **** p<0.0001.

Comparison of neutrophil A1 (CD63^lo^ CD16^hi^) and A2 (CD63^hi^ CD16^lo^) populations revealed significant differences in the surface expression of activation markers. The A2 population showed increased surface expression of secondary granule exocytosis (CD66b), in concordance with the canonical biological mechanisms of neutrophilic granule release, while surface CD14 was significantly higher in the A1 population (Fig. 2A). Interestingly, both A1 and A2 neutrophils in COVID-19 patients demonstrated surface expression of furin and ACE-2, suggesting the potential for interaction with SARS-CoV-2.

Next, given the transcriptional differences previously observed in A1 and A2 neutrophils in other airway diseases, we assessed how the transcriptional profile of A1 and A2 neutrophils relates to their impact on disease severity. To address this, we leveraged cell- and bacteria-free airway fluid from non-viral infectious acute lung injury patients that presented populations of both A1 and A2 neutrophils in their airways **(Fig. S3A)**, and used it in an i*n vitro* transmigration model ^18^ to generate A1 (transmigrated into LTB4) and A2 populations from normal peripheral blood neutrophils (transmigrated into the ALI/ARDS supernatant) **(Fig. S3B)**. A1 and A2 neutrophils differentiated *in vitro* showed similar activation profiles as those analyzed *in vivo*, **(Fig. S3C)**, as well as a distinct gene expression profiles **(Fig. S3D and S3E)**. A1 neutrophils displayed differential activation of inflammatory signaling pathways **(Fig. S3F)**, including the IL-17 signaling pathway, a major cytokine in neutrophilic-driven inflammation and mucosal immunity. In contrast, the A2 subset showed upregulation of antiviral pathways, most notably interferon signaling (Fig. 2B).

To assess the validity of the A2 neutrophil antiviral phenotype, we evaluated the single cell transcriptional dataset from 21 severe COVID-19 patients published by Bost and colleagues ^19^ **(Fig. S4A)**. After quality control filtering, the 21 samples were concatenated and 48,582 cells were recovered in total. Using the transcriptional profiles of *in vitro* A1 and A2, as well as expression of lineage-specific marker genes, we identified these two neutrophil populations in the single cell dataset from severe COVID-19 patients **(Fig. S4B)**. Of the cells sequenced by Bost and colleagues, we identified 25,664 neutrophils by expression of lineage-specific genes as FCGR3B, CXCR2, of which 20,600 w ere A1 (genes: CD177, S100A8, S100A9, PROK2) and 5,064 were A2 (genes: CD274, GBP4, GBP5, P2RY14, IFIT2, IFIT3, RSAD2) **(Fig. S4C and S4D)**. *In vivo* A2 neutrophils showcased a unique transcriptional profile that closely mirrored *in vitro* generated A2 neutrophils **(Fig. S4E)**, with increased expression of antiviral genes **(Fig. S4F)** belonging to many of the same antiviral pathways enriched *in vitro* (Fig. 2C). Furthermore, A2 neutrophils expressed genes involved in type I interferon signaling, both *in vivo* (Fig. 2D), and *in vitro* (Fig. 2E), with mean z-scores representing comparable upregulation of the type I interferon pathway in A2 neutrophils, suggesting a differential response upon SARS-CoV-2 encounter compared to A1 neutrophils.

### A2 neutrophils clear SARS-CoV-2

**Figure 3.**
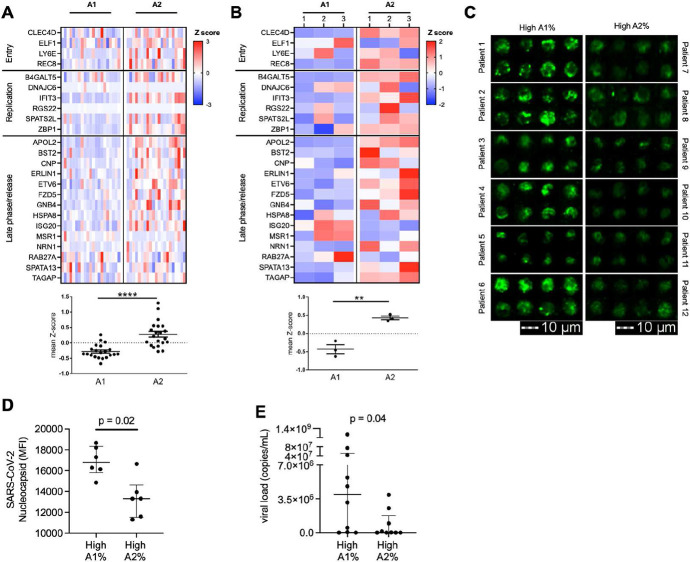
A2 neutrophils show differential anti-SARS-CoV-2 responses. **(A)** Expression analysis of genes implicated in SARS-CoV-2 intracellular antiviral response with mean z-score. Data were obtained from sc-RNA-seq and each column represents a patient (n=21 per group). **(B)** A1 and A2 neutrophils generated using an *in vitro* transmigration model showed differential gene expression for SARS-CoV-2 intracellular antiviral response. **(C)** Airway neutrophils from a subset of patients with high A1 or high A2 frequencies (n=6 per group) were stained for SARS-CoV-2 nucleocapsid (green) and acquired by image cytometry (see Fig. S3). **(D)** Patients with high A1% showed increased presence of intracellular SARS-CoV-2 in airway neutrophils. **(E)** Patients with high A1% showed increased presence of extracellular SARS-CoV-2 in the mBAL supernatant (n=19 patients). Results are shown as median and interquartile range. Statistical analysis was performed using Wilcoxon matched-pair signed rank’s test for paired analysis or the Wilcoxon rank-sum test for unpaired analysis.

We next assessed the capacity of A2 neutrophils to modulate SARS-CoV-2 infection. We investigated whether the A2 neutrophils from Bost et al with the antiviral profile were associated with genes known to have an impact on the SARS-CoV-2 lifecycle ^20^. Indeed, genes involved in the antiviral response to SARS-CoV-2 were upregulated in A2 neutrophils compared to A1 *in vivo* (Fig. 3A). The observed transcriptional changes matched the gene profile of *in vitro* A2 neutrophils (Fig. 3B), and included interferon-stimulated genes (ISG), suggesting a functional antiviral role.

To determine if the transcriptional changes observed in A2 neutrophils were related to alteration of viral loads *in vivo*, we measured SARS-CoV-2 presence in airway neutrophils using image cytometry **(Fig. S5)**. Patients with high A1 frequencies had increased intracellular viral staining in airway neutrophils compared to patients with high A2 frequencies (Fig. 3C and 3D), as well as lower viral copies in the extracellular milieu (Fig. 3E), suggesting different disease dynamics when the A2 population is predominant, including differential interaction with SARS-CoV-2 between the two neutrophil populations.

**Fig. 4.**
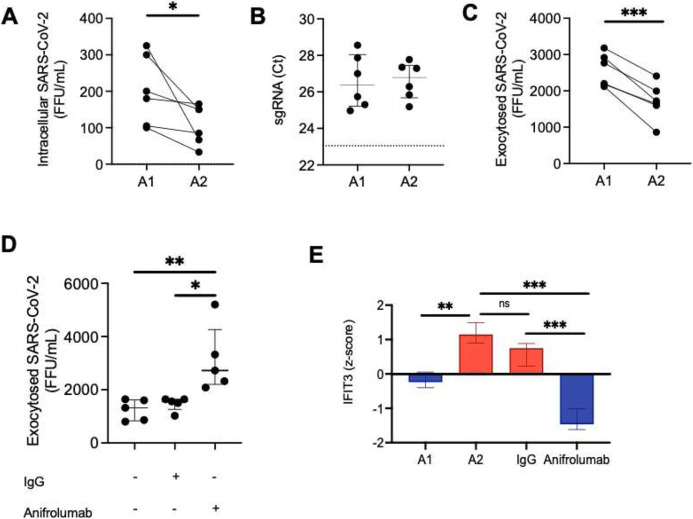
A2 neutrophils contribute to clearance of SARS-CoV-2. **(A)** A1 and A2 neutrophils incubated with SARS-CoV-2 (MOI = 1), with A2 neutrophils had reduced intracellular virions of infectious SARS-CoV-2 (n=6 neutrophil donors). **(B)** Subgenomic RNA (sgRNA) detection by RT-PCR of SARS-CoV-2 in A1 and A2 neutrophils (dotted line represents positive control, n = 6 neutrophil donors). **(C)** A1 and A2 neutrophils show differential exocytosis of infectious SARS-CoV-2 (n=6 neutrophil donors). **(D)** Type I interferon blockade with Anifrolumab increased exocytosis of infectious SARS-CoV-2 in A2 neutrophils (n=5 neutrophil donors) compared to IgG control or media alone. **(E)** IFIT3 expression by RNASeq. (FFU = foci forming units). Data are shown as median and interquartile range. Statistical analysis was performed using Wilcoxon matched-pair signed rank’s test or ANOVA with Tukey’s test for multiple comparisons. * p<0.05; ** p<0.01; *** p<0.001; n.s.: not significant.

Next, to address how neutrophils may influence viral dynamics, A1 and A2 populations were generated *in vitro* and then incubated with SARS-CoV-2 for 1 hour at an MOI of 1, followed by a 24 hours incubation after removal of the extracellular virus. Viral uptake was quantified at 1 hour by measuring extracellular unabsorbed virus. We observed that the uptake of SARS-CoV-2 did not differ between A1 or A2 neutrophil populations **(Fig. S6A)**, measuring similar amounts of extracellular SARS-CoV-2 in the media of A1 and A2 populations. Further, conditioned media from A1 and A2 populations were incubated directly with virus resulting in no effect on SARS-CoV-2 infectivity **(Fig. S6B)**. Likewise, conditioned media placed on VERO cells did not alter the susceptibility of epithelial cells to SARS-CoV-2 infection **(Fig. S6C and S6D)**. Next, we investigated whether A2 neutrophils *in vitro* had lower viral load intracellularly as previously observed *in vivo* by image cytometry. Indeed, we found that A2 neutrophils had reduced amount of infectious virus intracellularly as compared to A1 neutrophils (Fig. 4A) in accordance with the observed viral staining by image cytometry (Fig. 3C). In both populations viral replication was low (Fig. 4B) and there was no detectable difference between the two neutrophil subsets. Interestingly, A2 neutrophil populations were also found to have reduced exocytosis of infectious SARS-CoV-2 (Fig. 4C), pointing towards differential antiviral functions and viral clearance. Having observed the upregulation of genes in the type I interferon pathway and of interferon stimulated genes in the A2 population, we blocked type I interferon signaling in A2 neutrophils with a therapeutic monoclonal antibody (Anifrolumab) ^21^ targeting IFNAR and assessed viral clearance by the A2 neutrophil subset. Notably, type I interferon blockade with Anifrolumab increased exocytosis of infectious SARS-CoV-2 compared to IgG control or media alone conditions (Fig. 4D), showcasing a functional role of the type I interferon pathway in A2 neutrophils. Among the genes known to interfere with SARS-CoV-2 the only one that was significantly affected by the type I interferon blockade was the interferon-induced protein with tetratricopeptide repeats 3 (IFIT3 or ISG60) (Fig. 4E).

**Fig. 5:**
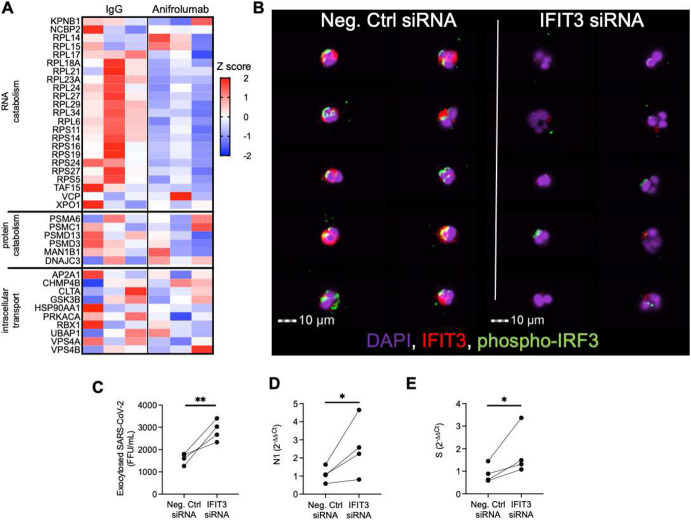
IFIT3 signaling modulates viral clearance in A2 neutrophils **(A)** Expression of genes in the macromolecule catabolic processes (GO: 0009057). **(B)** Image cytometry analysis of airway neutrophils for IFIT3 (red) and phospho-IRF3 (green) expression. (DAPI, purple). **(C)** IFIT3 knockdown increased exocytosis of infectious SARS-CoV-2 in A2 neutrophils (n=4 neutrophil donors). **(D-E)** IFIT3 knockdown modulates viral RNA catabolism (N1 and Spike protein RNA) in A2 neutrophils (n=4 neutrophil donors). (FFU = foci forming units). Data are shown as median and interquartile range. Statistical analysis was performed using Wilcoxon matched-pair signed rank’s test, * p<0.05; ** p<0.01.

IFIT3 has been shown to promote viral clearance in epithelial models of infection through activation of IRF3-mediated viral protein and RNA catabolism ^22^. However the presence and the mechanism of action of IFIT3 in airway neutrophils has not been elucidated. To better discern the role of IFIT3 in these cells, we first assessed whether catabolism-associate genes under the IRF3 transcriptional regulation (GO: 0009057), were modulated by blockade of type I interferon. Interestingly, treatment with Anifrolumab downregulated key IRF3-dependent catabolic genes (Fig. 5A). We next determined whether IFIT3 knockdown would affect these antiviral pathways in A2 neutrophils. Indeed, siRNA knockdown of IFIT3 **(Fig. S6E)** led to loss of IRF3 phosphorylation (Fig. 5B), required for nuclear translocation and transcription of catabolic genes. Further, while IFIT3 knockdown did not alter the ability to uptake SARS-CoV-2 **(Fig. S6F)**, it did reduce viral clearance (Fig. 5C) through the modulation of viral RNA catabolism (Fig. 5D-E). These results mirrored the alteration of viral clearance obtained upon treatment with Anifrolumab. Together, these findings showcase a novel mechanistic pathway of direct viral clearance in neutrophils dependent upon type I interferon signaling through IFIT3 expression.

## Discussion:

This study highlights a new type I interferon-dependent antiviral function of neutrophils in respiratory infections, which curbs COVID-19 immunopathology. Indeed, the loss of these antiviral neutrophils predicted poor clinical outcomes in severely ill COVID-19 patients with ARDS. Further, the identification of this unique cell population provides a novel avenue for cell-directed therapeutics.

Neutrophils have also been identified in other respiratory viral infections, although their roles remain relatively underappreciated. Previous studies focusing on the Respiratory Syncytial Virus (RSV) and Influenza A virus (IAV) showed that both viruses can be opsonized by the surfactant protein D and phagocytosed by neutrophils ^23,24^, and that RSV can undergo transcription in the neutrophil themselves ^25^, but a role of these cells in direct viral suppression was not observed. Further, depletion of neutrophil in vivo upon challenge with IAV led to severe lung pathology and mortality outcomes ^26,27^. Relatedly, we recently found that neutrophil populations in cystic fibrosis also undergo transcriptional changes ^17^, highlighting the potential plasticity of these cells in the lung microenvironment.

Prior studies have suggested that loss of type I interferon activity is detrimental to viral clearance in severe COVID-19 patients ^28,29^, but the relative cell-type contributions to this signaling in the lung is not well known ^30^. The identification of type I interferon signature in the A2 neutrophil population provides a discrete pathway by which these cells directly participate in viral clearance. Importantly, by demonstrating loss of virus in A2 neutrophils and then blocking this effect with inhibition of type I interferon signaling, we identified a new and important mechanism of viral clearance by these innate immune cells. Further, IFIT3 emerged as a potential key regulator of such antiviral functions, as it has previously been shown in other immune cells during viral infections ^31,32^. Although the mechanism of action for this protein in airway neutrophils remains poorly understood, our work shows that IFIT3 acts as a critical effector in neutrophil-related viral clearance through IRF3-dependent catabolic targeting of viral RNA. Examination of these neutrophils in other respiratory viral infections would provide further insight to how neutrophils may be contributing to antiviral immunity in these disorders.

Limited understanding of the immune profile in both systemic and lung compartments in ARDS remains an impediment to the development of appropriate disease-related biomarkers and therapeutics. The present study highlights the largest concomitant analysis of matched blood and airway immune landscapes in COVID-19 patients admitted to the intensive care unit and provides longitudinal analysis of matched matrices using multiparametric flow cytometry. The limited systemic and local modulation of innate immune responses over time suggests that the innate immune landscape and activation present at the onset of ARDS symptoms establish a clinical course of disease. This observation provides an approach to stratify the critically ill patient population, identifying COVID-19 ARDS patients at high risk for death early in ICU course for close clinical monitoring and early clinical trial recruitment.

To our knowledge, this study provides the first evidence of an antiviral neutrophil subset. Presence of this neutrophil subset was also detected in non-COVID ARDS, suggesting a broader role for this subpopulation and warranting more robust immunologic phenotyping in clinical conditions to better discern and inform therapeutics ^33^, including cell-based therapies ^34^. We observed A2 neutrophils frequencies as high as 70% of the total immune cells in the lung, therefore their impact on viral immunity is likely profound. Therapeutic considerations of this cell subset may potentially impact outcomes in both ARDS and viral lung disease.

## Online Methods

### Human subjects

Patients were recruited the Medical Intensive Care Unit (MICU) at the University of Alabama Hospital and at the Brigham and Women’s Hospital (Boston, Massachusetts). Patients included in the study were intubated, had confirmed SARS-CoV-2+ infection, and met the clinical definition of ARDS via Berlin criteria ^35^. Patient demographics are illustrated in Table S1. Control acute lung injury (ALI) mBAL were obtained from patients presenting with non viral infectious ALI. Demographics for ALI control group and healthy blood donors are shown in Table S2 and Table S3, respectively. All data and samples were collected in accordance with the University of Alabama at Birmingham’s IRB (COVID Enterprise IRB - IRB 300005127, IRB-300005209) and at the Brigham and Women’s Hospital (Boston, Massachusetts) (IRB- 2008P000495 and IRB- 2020P000447). Written consent was obtained prior to participation.

### H441 cell line

H441 cells (ATCC, Cat# HTB-174) were cultured in DMEM/F-12 media supplemented with 10% FBS (Corning), 2mM glutamine (Sigma), and 100 U/mL-0.1mg/mL penicillin/streptomycin (Sigma). 2.5 x10^5^ H441 were harvested at passages 2-3 and cultured at air-liquid interphase on the Alvetex scaffold (Reprocell) coated with Rat tail collagen (Sigma) as previously described ^36^. The basolateral medium DMEM/F-12 media supplemented with 2% Ultroser G (Crescent Chemical Co), 2mM glutamine (Sigma) and 100 U/mL-0.1mg/mL penicillin/streptomycin (Sigma) was replaced every two days. After 14 days the membranes were inversed to allow neutrophil loading on the basolateral side and apical migration as described below.

### Vero E6 cell line

Vero E6 cells (ATCC, Cat# C1008) were cultured in 96-well plates until confluence in Eagle’s MEM with 4% FBS (Denville Scientific Inc) supplemented with anti-biotic/anti-mycotic (Gibco) containing 100 units/mL of penicillin, 100 μg/mL of streptomycin, and 0.25 μg/mL of Gibco Amphotericin B.

### SARS-CoV-2 viral stocks

The original SARS-CoV-2 isolate USA-WA1/2020 was obtained from BEI resources (#NR-52281) and further propagated in Vero E6 cells through 2 more passages to obtain a working stock of virus at sufficient titer. A focus forming assay was used to quantify the titer of viral stocks and virus obtained from subsequent experimental tests.

### Sample collection and processing

Blood and mini-bronchoalveolar lavages (mBAL) were collected from COVID-19 patients at the time of admission to the MICU (N =52) and a subset 1 week afterwards (N=28). Blood was collected in citrate tubes by venipuncture, cells and plasma were separated by 400g, 10 minutes, 4°C centrifugation. The cellular fraction was resuspended in PBS-EDTA (2.5mM) to match the collection volume and stained for flow cytometry analyses. mBAL was collected by instillation of three separate 10-ml aliquots into the endotracheal tube via 14 Fr in-line suction catheter and the sample is aspirated back between each aliquot. Collected aspirate was mechanically dissociated on ice using an 18G needle and syringe. Airway immune cells were recovered after an 800g, 10 minutes, 4°C centrifugation, washed with PBS-EDTA (2.5mM) and stained for flow cytometry.

### Flow cytometry

Multiparametric flow cytometry analysis of whole blood and airway cells was standardized across study visits using the acquisition setting automatic calibration built into the BD software on the BD FACS Symphony instrument which provides constant and robust output from the flow cytometer over time. Samples were pre-stained for 10 minutes on ice in the dark with the Human TruStain FcX Fc blocking solution and the Zombie near IR reagent (Biolegend), then stained for surface markers (see **Table S4**). Cells were washed, fixed in Lyse/Fix Phosflow (BD Biosciences) and acquired on a BD FACS Symphony (BD Biosciences). Analysis and compensation were performed in FlowJo V10.6.2 (BD Biosciences).

### Image cytometry

Airway cells were fixed in BD Lyse/Fix Phosflow (BD Biosciences) and stored at −80 °C until use. Cells were thawed and washed with PBS-EDTA and permeabilized with Perm Buffer I (BD Biosciences) for 15 minutes at room temperature. Staining was performed in Perm Buffer I with DAPI (nuclear stain, 1*μ*M), cholera toxin B-Alexa Fluor 555 (to distinguish neutrophils which contain higher amount of lipid rafts than other immune cells, Thermofisher, 0.1 *μ*g) ^15^, as well as antibodies targeting CD63-APC (Biolegend, clone: H5C6, 0.2*μ*L), SARS-CoV-2 nucleocapsid conjugated with FITC (GeneTex, GTX135361, 3*μ*g), IFIT3 (Thermofisher, 1:100, RRID: AB_11153289), and phospho-IRF3 (Thermofisher, 1:100, RRID: AB_2532786). Cells were washed twice with Perm Buffer I and resuspended in PBS-EDTA prior to acquisition. Specimens were acquired on the Amnis Imagestream X Mark II (Luminex Corporation), with 40x magnification and low flow rate/high sensitivity on the INSPIRE software. Brightfield was set on channels 01 and 09, while scattering was set in channel 06. Data were analyzed using the IDEAS software v6.1 (Luminex Corporation).

### In vitro transmigration

ALI/ARDS mBAL supernatant was obtained by mechanical dissociation on ice using an 18G needle and syringe, followed by differential centrifugation at 800g and 3,000g to obtain the cell- and bacteria-free supernatant.

Neutrophils were isolated from whole blood using the density gradient Polymorphprep (Cosmo Bio USA) following manufacturer protocol ^37^. Neutrophil activation, purity (99% purity was obtained for every isolation), and viability was assessed using flow cytometry. Purified blood neutrophils were loaded on the basolateral side of the *in vitro* transmigration model as previously described ^36^ and allowed to migrate into the chemoattractant LTB_4_ (100nM, Sigma) (A1 neutrophils, ^17^) or into ALI/ARDS mBAL supernatant diluted 1:1 in plain RPMI (A2 neutrophils). Neutrophils were then collected at 14 hours post-transmigration (all conditions had at least 90% viability), washed, phenotyped by flow cytometry and used for downstream assays. Neutrophil conditioned media was obtained by isolating the supernatant (800g, 10 minutes, 4°C centrifugation) from transmigrated A1 or A2 neutrophils incubated in fresh RPMI for 3 hours post-transmigration.

### Type I interferon blockade

A2 neutrophils were transmigrated, as described above, into ALI/ARDS mBAL supplemented with either 10*μ*g/mL of IgG control (Biolegend) or 10*μ*g/mL of Anifrolumab (anti-IFNAR1, Creative biolabs), as previously described ^38^. Treatment with IgG control antibody or Anifrolumab was continued at the same concentration for the first hour of incubation for SARS-CoV-2. Viral infectivity in presence of these antibodies was tested and did not differ from a control condition with virus alone.

### Extracellular viral clearance assays

To assess direct effect on SARS-CoV-2, neutrophil conditioned media was incubated with 125 FFU of SARS-CoV-2 (1:1 in volume) for 30 minutes at 35°C, 5% CO_2_, and then used for foci forming assays. To determine the presence of an indirect effect, neutrophil conditioned media was incubated with a monolayer of VERO cells (1:1 dilution in RPMI) for 4 or 24 hours. Then, VERO cells were washed with RPMI and 50 FFU of SARS-CoV-2 was added as detailed in the foci forming assay section. Infection rate was assessed by foci forming assays.

### Intracellular viral clearance assays

A1 and A2 neutrophils were incubated in plain RPMI with SARS-CoV-2 at a MOI of 1 for 1 hour at 37°C, 5% CO_2_. After incubation, neutrophils were separated from the supernatant after a 10 minutes, 500g centrifugation. The supernatant was layered on VERO cells for foci forming assay, while neutrophils were resuspended in DMEM/F-12 media supplemented with 2% of 0.1*μ*m filtered FBS and incubated for 24 hours at 35°C, 5% CO_2_. Neutrophil viability after 24 hour was assessed at 80-90% for all conditions. Neutrophils were spun at 500g, 10 mins and the supernatant was used to quantify exocytosed SARS-CoV-2 by foci forming assay. To determine intracellular viral loads, neutrophils were lysed by freezing at −80°C. Samples were then spun at 500g, 10 mins and the supernatant was used for foci forming assays.

### Foci forming assay

Virus was serially diluted and added to the wells (100μL), and infection allowed to proceed for 1 hour on the Vero cells at 35°C. At the completion of the 1-hour incubation, an overlay of Eagle’s MEM with 4% FBS and antibiotics and was added to the inoculum on the cell monolayers such that the final volume was 200 μL per well. The infection was allowed to proceed for 24 hr. The next day, each plate was fixed by submerging the entire plate and contents in 10% formalin/PBS for 24 h. Detection of virus focal units, expressed as foci forming units (FFU) per mL, was performed on fixed 96 well plates. Briefly, plates were rinsed in H_2_O, and methanol:hydrogen peroxide (5% H_2_O_2_ in absolute methanol) added to the wells for 30 min with rocking to quench endogenous peroxidase activity. After quenching, plates were rinsed in H_2_O to remove methanol and Blotto (Thermo Scientific; equivalent to 5% non-fat dried milk) was added to the wells as a blocking solution for 1 hour. For primary antibody detection, a SARS-CoV-2 Spike/RBD antibody (Rabbit, Polyclonal, SinoBiologicals) was added to Blotto and incubated on the monolayers for at least 1 hour. Plates were rinsed 5 times with PBS, and further incubated with a secondary antibody of goat anti-rabbit IgG conjugated to horseradish peroxidase (Boster Biological Technology Co.) in Blotto for 1 hour. Plates were rinsed once with 0.05% tween in 1X PBS followed by 5 washes in PBS. Peroxidase activity was detected by use of Impact DAB detection kit (Vector Labs) per manufacturer’s instructions. Foci are counted manually from the scanned image of each well or otherwise microscopically imaged and quantitated. For conditions where foci were too dense to count, quantification of FFU was performed by densitometric analysis using Image Studio v5.2.5 on scanned wells (LI-COR Biosciences). All steps involved in handling viable virus were performed in a BSL-3 facility.

### RNA isolation

RNA from non infected neutrophils was isolated using the Nucleospin RNA kit (Takara). RNA from infected neutrophils was obtained by use of the RNeasy^®^ Plus mini kit (Qiagen) according to manufacturer’s instructions. All steps involved in handling viable virus were performed in a BSL-3 facility.

### Bulk RNA-sequencing

RNA isolated from *in vitro* samples was sequenced on the Illumina NextSeq500 at 75bp paired-end with a target of 20 million reads per sample. FASTQ files were checked for quality and raw sequencing data was aligned to the human reference genome (GRCh38) using STAR (Version 2.5.2) and quantmode was used to generate raw transcript counts. Differential gene expression was determined using DESeq2, while pathway analysis was performed using Ingenuity Pathway Analysis (Qiagen).

### Single-Cell RNA-sequencing

FASTQ files of bronchioalveolar lavage fluid from 21 severe COVID-19 patients from Bost et al ^19^ were downloaded from the European Nucleotide Archive (PRJNA661032). Transcriptomic alignment, barcode demultiplexing, and gene count quantification were done using Cell Ranger (version 4.0.0) with the force-cells option set to 10,000. The reference transcriptome was GRCh38 and was downloaded from 10X Genomics. All downstream analyses were done using Scanpy (version 1.6.0), a Python-based suite of packages for scRNA-seq analysis. For quality control filtering, cells were removed that contained <500 reads and <500 genes. Additionally, mitochondrial and ribosomal gene percentage cutoffs of >40% and >50%, respectively, were used to further eliminate low quality cells. Lastly, Scrublet (version 0.2.1) was used to remove potential doublets. After quality control filtering, the 21 samples were concatenated and 48,582 cells were recovered in total. Normalization was performed using Scran (version 1.10.2), and Scanpy was then used to perform complete cell cycle regression using the cell cycle genes identified by Tirosh and colleagues ^39^. Scanpy was used to select the top 1,500 highly variable genes, which were then used to calculate the top 13 principal components (PCs). Batch correction was performed using Harmony with the Scanpy external application programming interface (API; **Figure S4**). One categorical covariate was used for Harmony integration designating individual patient samples. Final dimensionality reduction was done using uniform manifold approximation and projection (UMAP) with default settings. Clustering was performed using the Leiden algorithm with a resolution of 0.3 followed by subclustering, and 8 clusters were identified. The assignment of cluster identities was guided by the expression of lineage-specific marker genes (**Figure S4**). Differential gene expression analysis was performed on normalized expression data using MAST (Seurat API).

### Viral RNA and viral replication

SARS-CoV-2 N1 and S RNAs in A2 neutrophils, as well as replication in A1 and A2 neutrophils was assessed by RT-PCR, with a positive control generated from a mix of clinical SARS-CoV-2 positive samples. The following primer sequences were used: Replication - Forward = CTCTTGTAGATCTGTTCTCTAAACGAAC, Reverse = GGTCCACCAAACGTAATGCG;

N1 – Forward = GACCCCAAAATCAGCGAAAT,

Reverse=TCTGGTTACTGCCAGTTGAATCTG;

S – CTCTTGTAGATCTGTTCTCTAAACGAAC,

Reverse = GGTCCACCAAACGTAATGCG;

RP – Forward = AGATTTGGACCTGCGAGCG, Reverse = GAGCGGCTGTCTCCACAAGT.

Primers were used at a final concentration of 200nM with Power SYBR^®^ Green RNA-to-CT^™^ 1-Step Kit (Thermofisher). Reverse transcription was performed at 48°C for 30 minutes, followed by activation at 95°C for 10 minutes. Amplification was carried out over 40 cycles as follow: denaturation (95°C, 15 seconds), annealing/extension (58°C, 1 minute). The melting curve was measured as follow: denaturing (95°C, 15 seconds), annealing (60°C, 15 seconds), denaturing (95°C, 15 seconds).

### IFIT3 knockdown

A2 neutrophils were transfected with 10 pmol of IFIT3 siRNA (s7155, Thermofisher) or of *Silencer*^™^ Cy^™^3-labeled Negative Control No. 1 siRNA (Thermofisher) using Lipofectamine RNAiMAX (Thermofisher) in Opti-MEM I reduced serum medium (Thermofisher) as per manufacturer’s protocol for 3 hours prior to the incubation with SARS-CoV-2. Knockdown efficiency was quantified by RT-PCR using the following primers: IFIT3 - Forward = GAACATGCTGACCAAGCAGA; Reverse = CAGTTGTGTCCACCCTTCCT ^40,41^; β-actin – Forward = AAAGACCTGTACGCCAACAC, Reverse = GTCATACTCCTGCTTGCTGAT. Primers were used at a final concentration of 200nM with Power SYBR^®^ Green RNA-to-CT^™^ 1-Step Kit (Thermofisher). Reverse transcription was performed at 48°C for 30 minutes, followed by activation at 95°C for 10 minutes. Amplification was carried out over 40 cycles as follow: denaturation (95°C, 30 seconds), annealing (58°C, 1 minute), extension (72°C, 30 seconds). The melting curve was measured as follow: denaturing (95°C, 15 seconds), annealing (60°C, 15 seconds), denaturing (95°C, 15 seconds).

### Statistical analysis

Statistical analyses were performed in JMP Pro 15 (SAS Institute), while graphing was done using Prism v8 (GraphPad) and R. Clustering analysis on concatenated samples using UMAP were created in FlowJo V10.6.2 (BD biosciences) using CD45, CD66b, CD16, CD14, CD63, HLA-DR and NE, with Euclidian method (nearest neighbor = 15, minimum distance = 0.5, number of components = 2). Threshold for A2 frequencies was determined by partitioning analysis followed by ROC curve for mortality (area = 0.64; A2 neutrophil % less than 42 defined as “low A2”). All data were analyzed using non parametric statistics: paired comparisons for each individual between two time points were done using Wilcoxon matched-pair signed rank’s test, non-paired analyses were computed using Wilcoxon rank-sum test, Fisher’s exact test was performed on contingency tables, and correlations were tested using Spearman’s Rho. Data are shown as median and interquartile range. P <0.05 was considered significant. Details can be found in the figure legends.

## Data Availability

Data that support the findings of this study have been deposited in Mendeley DOI: TBD.
